# Therapeutic Effects of Prolonged Cannabidiol Treatment on Psychological Symptoms and Cognitive Function in Regular Cannabis Users: A Pragmatic Open-Label Clinical Trial

**DOI:** 10.1089/can.2017.0043

**Published:** 2018-03-01

**Authors:** Nadia Solowij, Samantha J. Broyd, Camilla Beale, Julie-Anne Prick, Lisa-marie Greenwood, Hendrika van Hell, Chao Suo, Peter Galettis, Nagesh Pai, Shanlin Fu, Rodney J. Croft, Jennifer H. Martin, Murat Yücel

**Affiliations:** ^1^ School of Psychology and Illawarra Health and Medical Research Institute, University of Wollongong, Wollongong, Australia.; ^2^ The Australian Centre for Cannabinoid Clinical and Research Excellence (ACRE), New Lambton Heights, Australia.; ^3^ Brain and Mental Health Laboratory, Monash Institute of Cognitive and Clinical Neurosciences, School of Psychological Sciences, Monash University, Clayton, Australia.; ^4^ Discipline of Clinical Pharmacology, School of Medicine and Public Health, University of Newcastle, Callaghan, Australia.; ^5^ Graduate School of Medicine and Illawarra Health and Medical Research Institute, University of Wollongong, Wollongong, Australia.; ^6^ Centre for Forensic Science, University of Technology Sydney, Ultimo, Australia.

**Keywords:** cannabidiol, cannabis, attention, memory, psychological symptoms

## Abstract

**Introduction:** Chronic cannabis use has been associated with impaired cognition and elevated psychological symptoms, particularly psychotic-like experiences. While Δ^9^-tetrahydrocannabinol (THC) is thought to be primarily responsible for these deleterious effects, cannabidiol (CBD) is purported to have antipsychotic properties and to ameliorate cognitive, symptomatic, and brain harms in cannabis users. However, this has never been tested in a prolonged administration trial in otherwise healthy cannabis users. Here, we report the first study of prolonged CBD administration to a community sample of regular cannabis users in a pragmatic trial investigating potential restorative effects of CBD on psychological symptoms and cognition.

**Materials and Methods:** Twenty frequent cannabis users (16 male, median age 25 years) underwent a 10-week open-label trial of 200 mg of daily oral CBD treatment, while continuing to use cannabis as usual. The majority of participants were daily cannabis users who had used cannabis for several years (median 5.5 years of regular use). Participants underwent psychological and cognitive assessments at baseline (BL) and post-treatment (PT) and were monitored weekly throughout the trial.

**Results:** CBD was well tolerated with no reported side effects; however, participants retrospectively reported reduced euphoria when smoking cannabis. No impairments to cognition were found, nor were there deleterious effects on psychological function. Importantly, participants reported significantly fewer depressive and psychotic-like symptoms at PT relative to BL, and exhibited improvements in attentional switching, verbal learning, and memory. Increased plasma CBD concentrations were associated with improvements in attentional control and beneficial changes in psychological symptoms. Greater benefits were observed in dependent than in nondependent cannabis users.

**Conclusions:** Prolonged CBD treatment appears to have promising therapeutic effects for improving psychological symptoms and cognition in regular cannabis users. Our findings require replication given the lack of a placebo control in this pragmatic trial, but suggest that CBD may be a useful adjunct treatment for cannabis dependence.

## Introduction

The global trend toward legalization of cannabis for medicinal and recreational use highlights an urgent need for scientific investigation of the potentially harmful and beneficial effects of its constituent compounds, particularly in light of ongoing concerns regarding cannabis exposure effects on health.^1,2^ Prolonged frequent use of cannabis, particularly of high potency, has been associated with deleterious effects on psychological function, including increased risk of developing psychosis,^3,4^ impaired cognition,^5,6^ and alterations to brain structure^7^ and function.^8,9^ These adverse outcomes have been associated with the action of Δ^9^-tetrahydrocannabinol (THC), the primary psychoactive constituent of cannabis, and partial agonist at central cannabinoid (CB1) receptor sites.^10^ In contrast, the second most abundant constituent within cannabis, cannabidiol (CBD), is thought to have a broad range of therapeutic properties, including amelioration of the adverse psychological and cognitive effects of THC.^11^ Unlike THC, CBD is a low-affinity CB1 and CB2 receptor ligand and negative allosteric modulator of CB1, which reduces the binding of CB1 agonists, while augmenting endocannabinoid tone in an indirect manner.^12,13^

Therapeutic effects of CBD have been reported across a range of study designs and in different populations. In studies examining naturalistic exposure to CBD through hair analysis in regular cannabis users, greater concentrations of CBD have been associated with better cognitive performance, especially memory,^14^ fewer psychotic symptoms,^15,16^ and increased gray matter in the hippocampus.^17^ We recently demonstrated that naturalistic exposure to CBD in cannabis users is associated with normal hippocampal volumes relative to users exposed to THC, but not CBD.^18^ This suggests that CBD may be neuroprotective, perhaps through its role in synaptic plasticity and/or neurogenesis. Animal studies have also shown CBD to reverse THC-induced spatial memory deficits,^19^ conditioned place aversion,^20^ and decreased social interaction (for a review, see Refs.^12,21^), and importantly, to increase hippocampal cell survival and neurogenesis.^22^ Administration of pure compounds to humans showed that CBD produces opposite effects to THC in the nature of regional brain activation^23^ and acute exposure to CBD ameliorates cognitive and psychotic-like symptoms induced by THC in cannabis users.^24^ Despite promising evidence of the therapeutic effects of CBD, no study to date has examined the potentially restorative effects of prolonged CBD administration to cannabis users.

This study is the first investigation of potential therapeutic effects of prolonged daily administration of CBD to regular cannabis users using a pragmatic open-label design, wherein cannabis users maintained their naturalistic use of cannabis. On the basis of reviewed literature, we focused on symptoms of depression, trait anxiety, and psychosis-proneness,^4,25,26^ and cognitive performance within the domains of attention/executive function and learning and memory, being the most sensitive to the deleterious effects of chronic cannabis use.^5^ We hypothesized that prolonged administration of CBD would improve psychological functioning and cognitive performance in regular cannabis users. We had no *a priori* hypothesis regarding the effects of prolonged CBD exposure on ongoing patterns of cannabis use, since potentially diminished effects of THC could either result in a reduction or an increase in cannabis use if its rewarding effects are mitigated.

## Materials and Methods

### Participants

Twenty cannabis users, recruited by advertising, participated in this ∼10-week pragmatic open-label clinical trial. Inclusion criteria required participants to have used cannabis at least monthly for 6 months (the majority vastly exceeded this criterion; Table 2). Following a telephone screen and a subsequent face-to-face semistructured interview (as previously used in our studies^27,28^), participants were excluded for any lifetime head injuries requiring hospitalization, neurological conditions, current psychiatric medication, and current psychiatric diagnoses (personal, or first-degree relative for psychotic disorders) or alcohol dependence assessed using the Mini-International Neuropsychiatric Interview—MINI Plus.^29^ Participants were also excluded based on self-reported history of regular other illicit drug use (>once/month for >6 months in the past 3 years); occasional recreational use (<once/month) was not an exclusion criterion. Participants were not required to alter their usual patterns of cannabis use; however, they were requested to abstain from other drug use throughout the trial (self-report corroborated by weekly urine drug screen) and from cannabis and alcohol for at least 12 h before baseline (BL) and post-treatment (PT) test sessions. Participants were familiarized with study procedures before providing written informed consent at each testing session and received incremental reimbursements for participation (completion total of AU$650). The study was approved by the University of Wollongong and Illawarra Shoalhaven Local Health District Health and Medical Human Research Ethics Committee and registered as a clinical trial (ISRCTN89498802).

### Procedure

The trial was conducted over 12 weeks, comprising 2 days of BL assessments, 10 weeks (on average) of daily CBD administration, face-to-face weekly monitoring, and provision of CBD capsules, and minimum 12-h washout of CBD, cannabis, and alcohol before 2 days of PT assessments. The 2-day sessions occurred mostly consecutively (maximum 1 week apart; CBD administration maintained for PT sessions) and consisted of structured interview, and clinical, cognitive, electroencephalogram (EEG), and magnetic resonance imaging (MRI) assessments (EEG and MRI outcomes will be reported elsewhere). Specifically, beyond telephone screening, the BL assessment at the University included the following: (1) consent signing; (2) our customized semistructured interview^27,28^ to further assess demographic information, detailed history of current and previous cannabis and other licit and illicit substance use, and cannabis-specific measures of withdrawal (Cannabis Withdrawal Scale; CWS),^30^ dependence (Severity of Dependence Scale; SDS),^31–33^ and (retrospectively) experiences while intoxicated (Cannabis Experiences Questionnaire; CEQ)^34,35^; (3) the MINI Plus to screen for psychiatric disorders, and a range of scales to assess symptoms of depression, anxiety, mood, and psychosis liability, and global functioning (as primary outcome measures—see section, “Psychological symptom, cognitive and substance-related measures”); (4) the vocabulary and matrix subscales of the Wechsler Abbreviated Scale of Intelligence^36^ to estimate full scale IQ; and (5) height and weight were measured, and a blood sample and urine sample were obtained (see section “CBD administration and weekly monitoring”). Participants then proceeded to cognitive testing and the EEG session, with an MRI session on the second day. PT assessments were near identical, excluding only those assessments required to be taken once (e.g., detailed history and height). Follow-up telephone assessments occurred 1 week, 1 month, and 3 months after completion of the trial to monitor any withdrawal symptoms experienced as a result of ceasing CBD treatment, general physical and mental well-being, and any changes to substance use.

#### Psychological symptom, cognitive and substance-related measures

The primary outcomes of this study were psychological symptom and cognitive outcomes. Table 1 provides a full listing of study measures and their schedule of administration. To assess changes in psychological symptoms following CBD treatment, participants completed self-administered questionnaires related to depressive (Beck Depression Inventory; BDI^37^), anxiety (State-Trait Anxiety Inventory^38^; STAI-I state and STAI-II trait), and psychotic-like (Community Assessment of Psychic Experiences; CAPE^39^) symptoms at BL and PT, and overall functioning (Global Assessment of Functioning; GAF,^40^ and Social Occupational Functioning Assessment Scale; SOFAS^40^) was assessed by the researchers. Changes from BL to PT in cognitive function were assessed using the Rey Auditory Verbal Learning Test (RAVLT^41^; alternate forms), and the Attention Switching Task (AST), a task of executive function measuring cued attentional set-shifting from the Cambridge Neuropsychological Test Automated Battery (CANTAB Connect; iPad version). Changes from BL to PT were also assessed for cannabis and alcohol-related cognitions and behaviors (CWS, SDS, CEQ, and the Alcohol Use Disorders Identification Test; AUDIT^42^). Cumulative cannabis, alcohol, tobacco, and any other drug use were ascertained weekly using the Timeline Follow-Back procedure (TLFB).^43^ Participants were encouraged to report any observed (positive or negative) effect of CBD treatment through a semistructured interview.

**Table 1.** **Baseline, Weekly, and Post-Treatment Measures**

Measures	Baseline	Weekly sessions	Post-treatment
Biological samples			
Blood	√	√	√
Urine	√	√	√
Substance use related			
TLFB	√ (30 day)	√ (7 day)	√ (7 day)
CWS	√		√
SDS	√		√
CEQ	√		√
AUDIT	√		√
Clinical symptoms and overall functioning			
BDI	√	√	√
STAI-I and STAI-II	√	√-I only	√
POMS	√	√	√
BPRS		√	
CAPE	√		√
SPQ	√		√
GAF	√		√
SOFAS	√		√
Cognitive			
RAVLT	√		√
AST	√		√

The POMS, BPRS, and SPQ were administered for another study and are not reported here.

AST, Attention Switching Task (CANTAB Connect; Cambridge Cognition); AUDIT, Alcohol Use Disorders Identification Test^42^; BDI, Beck Depression Inventory^37^; BL, baseline; BPRS, Brief Psychiatric Rating Scale^50^ (no changes were observed across weekly sessions); CAPE, Community Assessment of Psychic Experiences^39^; CEQ, Cannabis Experiences Questionnaire^34,35^; CWS, Cannabis Withdrawal Scale^30^; GAF, Global Assessment of Functioning DSM-IV-TR^40^; POMS, Profile of Mood States^51^; PT, post-treatment; RAVLT, Rey Auditory Verbal Learning Test (administered as per Lezak, 2004 with alternate forms at BL and PT)^41^; SDS, Severity of Dependence Scale^31–33^; SOFAS, Social Occupational Functioning Assessment Scale DSM-IV-TR^40^; SPQ, Schizotypal Personality Questionnaire^52^; STAI-I and STAI-II: State-Trait Anxiety Inventory-I (state anxiety) and -II (trait anxiety)^38^; TLFB, Timeline Follow-back procedure.^43^

#### CBD administration and weekly monitoring

At BL and each weekly session, participants received 28 gelatin-coated capsules containing 50 mg of 99.5% pure crystalline CBD (of herbal origin) solved in Miglyol 812 and Softisan 378 (Trigal Pharma Ltd; a subsidiary of the BioSynthesis Pharma Group Ltd). Participants were instructed to swallow 4 (50 mg) capsules per day (2 in the morning and 2 in the evening, spaced to optimize steady-state plasma concentrations), equating to 200 mg/day CBD. This dose was selected as a “medium” level dose based on the range of therapeutic doses reported in human studies (e.g., ≥800 mg/day in psychotic individuals^44,45^), and for caution since no previous study had administered prolonged and relatively high doses of CBD to ongoing cannabis users. Participants received an SMS text message (morning and evening) reminding them to take their capsules. At each weekly session, participants returned any unused capsules and were given a new bottle containing 28 capsules for the following week. Adherence was measured by the number of capsules returned and participants reported the times of any missed doses. Heart rate and blood pressure were measured; no significant variations occurred over the course of the trial (data not reported). Blood samples and urine samples (for drug screens and pregnancy testing in females—an exclusion criterion) were taken weekly. Plasma was analyzed by LC-MS/MS for cannabinoid (CBD, THC, and THC metabolite) concentrations.^46^ Urine samples were subjected to ProScreen™ Dip Tests to corroborate self-reported abstinence from drugs other than cannabis. All BL and PT urine samples, and a random selection of samples provided weekly during the trial proceeded to urinary drug screen testing (total 98 urine samples analyzed; 5 samples per participant on average).

### Statistical analyses

Statistical analyses were conducted using SPSS 24.0. Primary outcome measures were change from BL to PT in BDI, STAI-II, and CAPE (positive, negative, and depressive psychotic-like symptoms) scores, in performance on the RAVLT and AST, and in cannabis, tobacco, and alcohol use measures. Significant change was assessed by paired sample *t*-tests, or Wilcoxon signed-rank tests for non-normally distributed data. Outcomes were further explored by group: heavy versus light users according to median split on lifetime occasions of use, and dependent versus nondependent users according to cutoff scores on the SDS (≥3)^47^ at BL, by repeated-measures ANOVA (rmANOVA) for time by group interactions (with covariates as required), or Wilcoxon signed-rank tests for skewed data. Spearman's correlations explored associations between BL and PT scores or change scores in psychological symptoms, cognitive performance, cannabis use measures, CBD dose consumed (self-report), and plasma CBD concentrations (mean, maximum, final week of trial, and assessment day).

## Results

### Participant characteristics, patterns of cannabis use, medication adherence, and plasma cannabinoid concentrations

Demographic and substance use measures for the overall sample are provided in Table 2. Participants were mostly young adult males (median age 25; 4 females) and the majority had completed some tertiary education. They were using cannabis on a median 25 days/month and had been using regularly for a median of 5.5 years. The majority refrained from other illicit drug use during the course of the trial, with one exception: one participant self-reported using LSD, ecstasy, ketamine, mushrooms, or mescaline on multiple occasions during the trial; none of these drugs were detected in urinary drug screens and the self-reported use was not alerted by the participant to the research team until the trial was completed. Since this was a naturalistic study of cannabis users in the community, and the participant was not an outlier on any measure, his data were retained in the analyses reported in this study.

**Table 2.** **Participant Demographics and Cannabis, Tobacco, and Alcohol Use Measures at Baseline and Post-Treatment**

	Baseline	Post-treatment	*t*/*Z*^a^	*p*	Effect size *d*/*r*^a^
*n*=20					
Age (years)	25.1 [20.6–46.8]	—			
Gender (M/F)	16/4	—			
Education (years)	15.5 [11–23]	—			
IQ	113.4 (10.08)	—			
BMI	22.68 (2.86)	—			
Tobacco use (cigarettes/month)^b^	9 [0–540]	21.5 [0–308]	0.86	0.39	0.19
Alcohol frequency (days/month)^b^	4 [0–30]	8 [0–21]	1.53	0.13	0.34
Alcohol quantity (std drinks/month)^b^	19 [0–102]	29.8 [0–128]	1.14	0.26	0.25
AUDIT	8.70 (5.36)	7.60 (4.87)	2.05	0.47	0.55
Cannabis use					
Age of first use (years)	17.34 (0.51)	—			
Age of onset regular use (years)	19.89 (0.47)	—			
Duration of regular use (years)^c^	5.17 [0.5–28.8]	—			
Duration of use lifetime	7.07 [4.2–31.8]	—			
Estimated lifetime occasions of use	1591 [141–8708]	—			
Past month frequency (days/30)	25.0 [2–30]	30.0 [3–30]	−1.15	0.14	−0.26
Past month quantity (cones)^d^	123.75 [9–1125]	105.0 [8–1080]	−0.57	0.57	−0.13
Cumulative quantity across the trial (cones)^d^	—	381 [9.5–2195]			
Time since last smoked (h)	17.17 [12–408]	17.25 [11.8–252]	1.92	0.055	0.43
CEQ					
Euphoria	43.75 (9.54)	38.65 (8.53)	4.12	0.001	0.93
Paranoid/dysphoric	36.75 (7.24)	35.25 (5.62)	1.21	0.24	0.28
After effects	21.85 (8.60)	20.45 (6.72)	1.03	0.32	0.24
Amotivation	15.10 (6.06)	14.35 (5.32)	0.79	0.44	0.18
Psychotic	6 [4–13]	6 [4–11]	−0.67	0.50	0.11
CWS	3 [0–29]	3 [0–32]	1.47	0.14	0.23
SDS	3.40 (2.37)	3.25 (2.17)	0.37	0.72	0.08

Mean (SD) or median [range].

^a^ Paired samples *t*-test for normally distributed data; Wilcoxon signed-rank test for skewed data.

^b^ From 30-day Timeline Follow-back.^43^

^c^ Duration of regular use in regular users only.

^d^ Cones used in waterpipe; three cones are equivalent to one standard sized joint.

BMI, body mass index; SD, standard deviation.

Dependent users did not differ from nondependent users in years of regular use or lifetime occasions of use (although the latter was marginal at *p*=0.054, with 8 heavy and 4 light users in the dependent group and 2 heavy and 6 light in the nondependent group). Dependent and nondependent users also did not differ in cumulative quantity of cannabis (cones), alcohol (standard drinks), and tobacco (cigarettes) consumed over the weeks of the trial (calculated from weekly TLFB interviews) (all *p*>0.08). Only cumulative cannabis use differed between heavy and light users, as to be expected (median 1064 vs. 120 cones, *p*=0.003; all other *p*>0.075).

The prolonged CBD treatment was well-tolerated with no adverse effects during the trial or in the follow-up period, including no withdrawal symptoms or changes in cannabis or other drug use after ceasing CBD treatment. Ten weeks of daily CBD treatment was planned and completed by most participants, missing only occasional doses. However, in this pragmatic community trial, a range of participant-related issues (e.g., work commitments and scheduling difficulties with PT appointments) resulted in one participant completing only 5.5 weeks of CBD treatment, while four participants continued to take CBD for 11–12 weeks to ensure PT sessions were conducted after a similar washout period for all participants (≥12 h after the last CBD capsule was consumed; median 15 h; range 12–23 h). Participants reported consuming a median of 258 capsules over the course of the trial (range 154–334), resulting in a total median dose of 12,900 mg CBD (range 7700–16,700 mg) over a median 10 weeks, with median adherence to consuming the 200 mg daily dose of 93.16% (range 68.67%–99.35%; median daily dose 200 mg; mean daily dose 184.05 mg, SD 13.99).

**FIG. 1.**
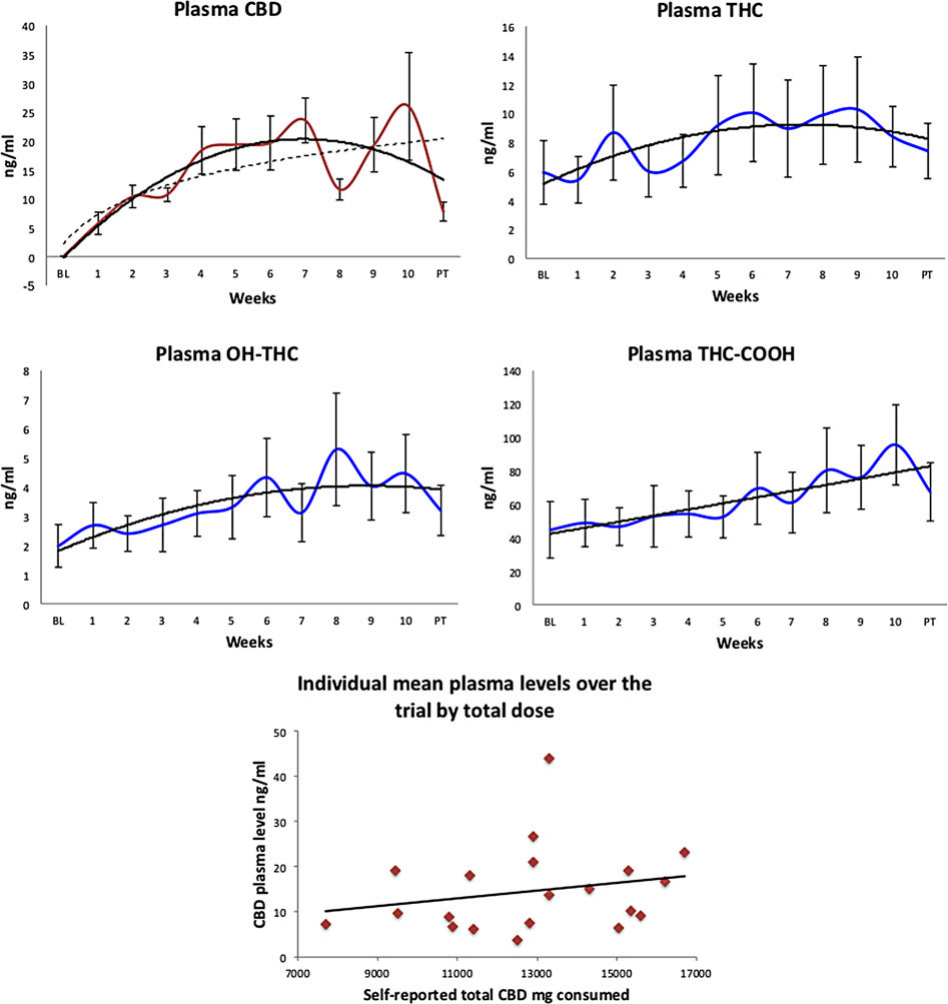
Plasma concentrations of CBD (from administration; dashed line logarithmic fit to indicate likely model without washout before PT), THC (from cannabis use external to the trial), THC-COOH and OH-THC (metabolites of cannabis used externally) depicted across 10 weeks of CBD treatment, and after an at least 12-h washout (PT) (solid line polynomial fit). Error bars indicate ±1 SE. Average plasma CBD concentration across the trial as a function of self-reported total dose (number of capsules) consumed by each individual (linear fit; *rho*=0.32, *p*=0.17). CBD, cannabidiol; PT, post-treatment; THC, Δ^9^-tetrahydrocannabinol.

Average plasma CBD concentrations across the trial are shown in Figure 1, demonstrating relative achievement of stable concentrations, although with much individual variability as expected for metabolism of this compound following oral administration. No CBD was detected in plasma at BL. Figure 1 also displays concentrations of plasma THC and THC metabolites, and Table 3 provides the weekly data across the trial for each compound. Interestingly, average weekly plasma CBD concentrations were positively correlated with average weekly plasma THC-COOH concentrations (*r*=0.65, *p*=0.017). Plasma CBD concentrations were not significantly correlated with self-reported dose of CBD consumed; Figure 1 nevertheless shows a trend toward a relationship. Plasma CBD concentrations did not differ between heavy and light cannabis users, but dependent users had significantly lower mean (10.11 ng/mL vs. 21.26 ng/mL; *p*=0.006) and maximum plasma concentrations (median 19.45 vs. 66.80 ng/mL; *p*=0.002) than nondependent users, despite not differing in self-reported total CBD dose consumed (*p*=0.97). Subsequent analyses of symptoms and cognition by group used rmANCOVA with mean plasma CBD concentrations and cumulative cannabis use measures from across the trial as covariates.

**Table 3.** **Mean, Median, and Standard Deviation, and Minimum and Maximum Plasma Concentrations of CBD, THC, THC-COOH, and OH-THC at Baseline, Across 10 Weeks of CBD Treatment, and After a ≥12-H Washout at Post-Treatment**

	BL	w1	w2	w3	w4	w5	w6	w7	w8	w9	w10	PT
CBD (ng/mL)												
Mean	0.1	5.8	10.4	10.7	18.4	19.4	19.7	23.6	11.6	19.4	26.0	7.8
Median	0.1	0.1	8.9	9.7	9.9	12.2	11.2	17.1	8.1	13.0	7.5	5.1
SD	0.1	8.6	8.7	5.3	18.3	19.8	20.9	17.4	8.1	21.1	41.6	7.4
Min	0.0	0.0	0.2	1.9	2.6	3.0	1.4	6.6	3.4	1.5	3.2	1.2
Max	0.3	27.9	36.6	21.4	65.8	59.8	76.5	65.3	32.7	70.8	129.5	31.0
THC (ng/mL)												
Mean	5.9	5.4	8.7	6.0	6.7	9.2	10.0	8.9	9.9	10.3	8.4	7.4
Median	1.9	1.4	3.6	2.0	2.5	4.5	2.4	1.7	3.6	2.3	6.2	2.7
SD	9.8	7.2	14.6	7.9	8.1	15.3	15.0	14.9	15.2	16.2	9.3	8.5
Min	0.0	0.0	0.0	0.0	0.0	0.0	0.0	0.0	0.0	0.0	0.0	0.0
Max	37.2	21.4	62.3	26.2	26.2	63.3	56.9	46.2	58.0	56.8	30.5	26.5
OH-THC (ng/mL)												
Mean	2.0	2.7	2.4	2.7	3.1	3.3	4.3	3.1	5.3	4.0	4.5	3.2
Median	0.9	1.0	1.5	0.9	1.4	1.7	1.3	1.2	1.6	1.6	2.2	1.3
SD	3.3	3.5	2.7	4.1	3.5	4.9	6.0	4.4	8.6	5.2	6.0	3.9
Min	0.0	0.0	0.0	0.0	0.0	0.0	0.0	0.0	0.0	0.0	0.0	0.0
Max	13.4	11.8	8.8	17.5	10.1	16.9	17.5	15.7	27.8	15.1	18.0	12.4
COOH-THC (ng/mL)												
Mean	44.7	48.8	46.7	52.8	54.2	52.5	69.5	61.0	80.2	76.1	95.5	67.4
Median	18.9	28.7	27.2	23.4	30.8	32.7	28.8	21.2	27.6	26.6	61.6	25.3
SD	75.5	63.2	50.5	82.1	61.5	56.2	96.2	80.9	113.2	85.3	107.1	77.7
Min	0.2	0.5	0.6	0.2	0.2	0.2	0.1	0.5	0.3	0.5	0.1	0.2
Max	315.5	199.1	155.0	352.8	196.9	158.4	358.0	227.4	454.8	203.1	289.7	251.4

BL, baseline; CBD, cannabidiol; Max, maximum; Min, minimum; PT, post-treatment; THC, Δ^9^-tetrahydrocannabinol; w1–w10, week 1–week 10.

Cannabis frequency and quantity measures did not change significantly from BL to PT (all *p*>0.14). However, there was a significant decrease in CEQ euphoria levels reported to be experienced after using cannabis (*p*=0.001 in the overall sample; no time by group interactions, *p*=0.005 after cumulative cannabis use included as covariate), and many participants self-reported feeling less high when they smoked cannabis over the course of the trial. No other CEQ measures were altered significantly from BL to PT, nor were measures of severity of dependence on cannabis (SDS) or withdrawal from abstaining for at least 12 h before each test session (CWS). There were no significant changes in tobacco or alcohol use from BL to PT. AUDIT scores marginally reduced overall (*p*=0.055) and significantly in dependent users (BL 9.08 vs. PT 7.25) relative to nondependent users (BL 8.13 vs. PT 8.13) (time by group: *p*=0.095; *p*=0.043 with covariates). SDS scores appeared to reduce in dependent users (BL 5.01 vs. PT 4.50) and increase in nondependent users (BL 0.88 vs. PT 1.38), but the interaction was not significant (*p*=0.20).

### BL to PT differences in psychological symptoms

**FIG. 2.**
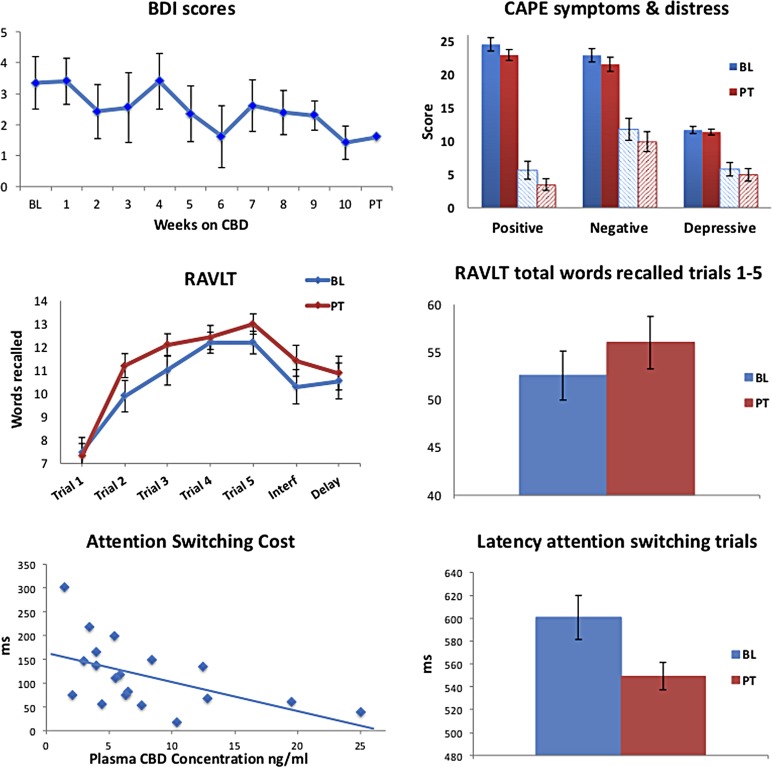
Psychological symptom scores and cognitive performance in the whole sample across the CBD treatment trial or at BL and PT: depressive symptoms (BDI scores) over the course of the trial; CAPE positive, negative, and depressive symptom frequency (solid) and distress (dashed) scores at BL and PT; RAVLT learning curves and total words recalled across trials 1–5 at BL and PT; CANTAB Attention Switching Task switching cost as a function of plasma CBD concentration on the day of testing (*rho*=−0.61, *p*=0.006), and latency during switching blocks at BL and PT. BDI, Beck Depression Inventory; BL, baseline; CAPE, Community Assessment of Psychic Experiences; RAVLT, Rey Auditory Verbal Learning Test.

Table 4 reports psychological symptom measures at BL and PT for the overall sample. Severity of depressive symptoms (BDI) was significantly lower at PT than at BL (*p*=0.017) (Fig. 2 depicts a linear decrease over the weeks of the trial; *r*=−0.78, *p*=0.003). Participants also reported significantly fewer positive psychotic-like symptoms (CAPE-positive symptom frequency, *p*=0.025) with lower levels of associated distress (CAPE-positive symptom distress, *p*=0.022) at PT. Reduction in positive symptom frequency remained significant after cumulative cones of cannabis smoked across the trial was included as a covariate (*p*=0.014). There were trends toward decreased overall symptom frequency and distress (CAPE total frequency, *p*=0.056; total distress, *p*=0.051). In contrast, state anxiety increased PT relative to BL (STAI-I, *p*<0.015). No changes were observed for trait anxiety, global or social, and occupational functioning (Table 4). These results suggest that prolonged CBD treatment may improve depressive and positive psychotic-like symptoms in regular cannabis users.

**Table 4.** **Psychological Functioning and Symptom Scores and Cognitive Performance at Baseline and Post-Treatment**

	Baseline	Post-treatment	*t*/*Z*^a^	*p*	Effect size *d*/*r*^a^
BDI	2.5 [0–14]	0.5 [0–12]	2.49	0.013	0.39
STAI-I	23.5 [20–38]	29.5 [20–49]	−2.43	0.015	0.38
STAI-II	32 [20–63]	32 [20–49]	1.19	0.23	0.19
GAF	85 [70–95]	85 [60–95]	0.88	0.38	0.14
SOFAS	85 [65–95]	85 [55–95]	0.88	0.38	0.14
CAPE					
Frequency total	59.25 (10.22)	56.00 (8.97)	2.21	0.040	0.50
Distress total	23.25 (16.49)	18.40 (12.50)	2.35	0.030	0.58
Negative frequency	22.95 (4.49)	21.6 (4.78)	1.62	0.12	0.36
Negative distress	11.80 (7.36)	9.95 (6.71)	1.71	0.10	0.39
Positive frequency	24.60 (4.49)	23.00 (3.68)	2.65	0.016	0.62
Positive distress	3 [0–20]	2 [0–15]	2.29	0.022	0.36
Depressive frequency	11.70 (2.39)	11.40 (1.96)	0.56	0.58	0.13
Depressive distress	5.80 (4.57)	4.95 (4.26)	0.96	0.35	0.22
RAVLT					
Words recalled Trials 1–5	52.55 (11.07)	56.00 (9.70)	−2.25	0.038	0.53
Recall postinterference	10.30 (3.29)	11.42 (2.95)	−2.30	0.033	0.54
Delayed recall	10.55 (3.35)	10.89 (3.25)	−0.84	0.41	0.19
AST					
Overall latency correct	518.65 (62.68)	492.60 (36.81)	2.44	0.025	0.63
Latency congruent	493.68 (56.96)	474.00 (38.68)	2.07	0.053	0.50
Latency incongruent	552.28 (68.49)	513.40 (42.55)	3.53	0.002	0.90
Latency switching trials	600.63 (86.21)	549.30 (53.40)	2.97	0.008	0.71
Switching cost	152.20 (70.80)	115.38 (68.78)	2.38	0.028	0.53

Mean (SD) or median [range].

^a^ Paired samples *t*-test for normally distributed data; Wilcoxon signed-rank test for skewed data.

No significant time by group interactions were observed on any measure when comparing groups of heavy and light users with or without cumulative cones smoked across the trial as a covariate. However, in nonparametric tests for non-normal variables, only heavy users showed a significant reduction in depressive symptoms (median BL 3.0 vs. PT 0.0; *Z*=2.53, *p*=0.011), while light users showed significant reduction in CAPE-positive symptom distress (BL 8.5 vs. PT 2.5, *Z*=2.05, *p*=0.041).

**FIG. 3.**
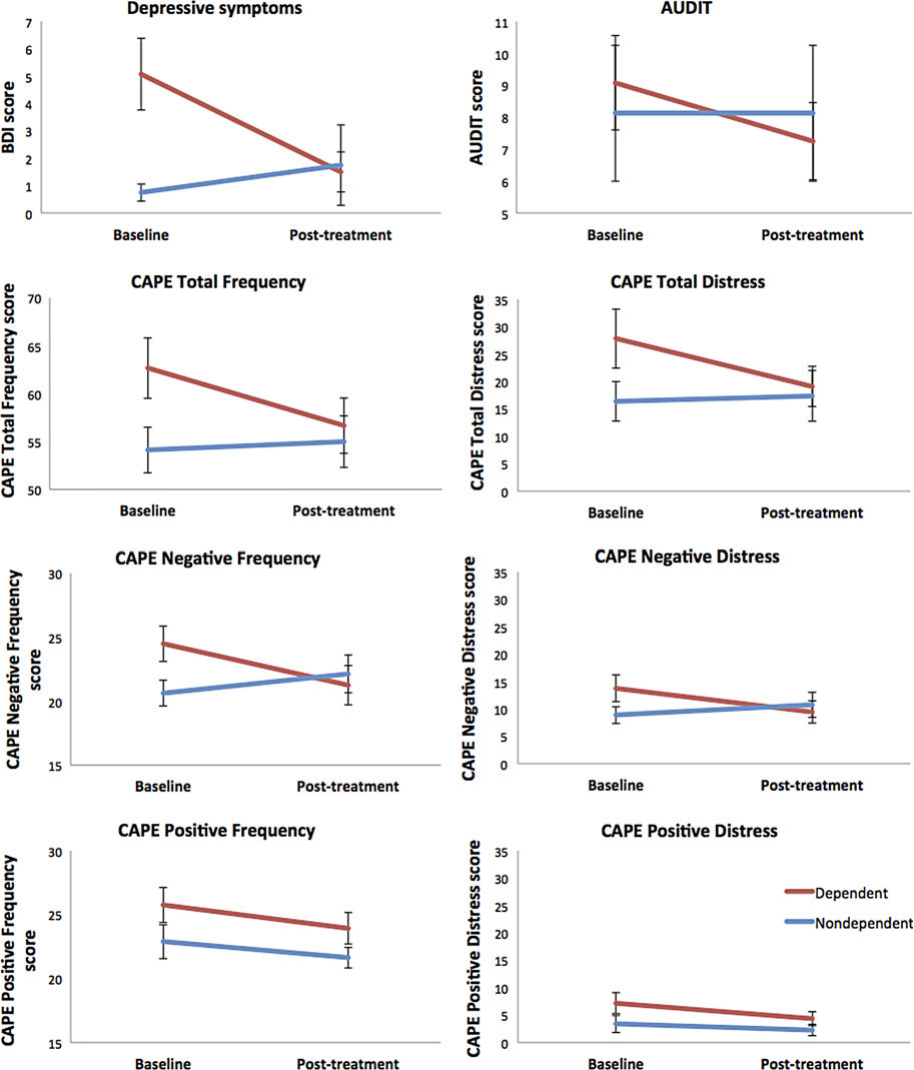
Psychological symptom changes from baseline to PT in dependent and nondependent cannabis users. Group-by-time interactions for BDI scores (*F*_1,18_=8.29, *p*=0.010); Alcohol Use Disorders Identification Test scores (*F*_1,18_=3.10, *p*=0.095); CAPE Total Symptom Frequency score (*F*_1,18_=6.82, *p*=0.018); CAPE Total Symptom Distress scores (*F*_1,18_=7.03, *p*=0.016); CAPE-negative symptom frequency scores (*F*_1,18_=12.64, *p*=0.002); CAPE-negative symptom distress scores (*F*_1,18_=12.83, *p*=0.002), and CAPE-positive symptom frequency scores (*F*_1,18_=0.21, *p*=0.649). Wilcoxon signed-rank Test for CAPE-positive symptom distress scores: dependent users *Z*=2.11, *p*=0.035; nondependent users *Z*=0.92, *p*=0.356.

Dependence status was far more sensitive to the effects of CBD treatment; significant time-by-group interactions were observed for CAPE total frequency (*p*=0.018; *p*=0.044 with covariates) and distress scores (*p*=0.016; *p*=0.026 with covariates), and CAPE-negative frequency (*p*=0.002; *p*=0.004 with covariates) and distress scores (*p*=0.002; *p*=0.007 with covariates), with dependent users showing greater reduction in symptoms than nondependent users (Fig. 3). CAPE-depressive symptom frequency scores showed a significant reduction from BL to PT (*p*=0.025), which did not interact with group. Dependent users showed significant reduction in BDI scores (median BL 4.0 vs. PT 1.0; *Z*=2.82, *p*=0.005) and CAPE-positive symptom distress (BL 5.5 vs. PT 3.0; *Z*=2.11, *p*=0.035), with no change observed in nondependent users. Furthermore, only dependent users showed an increase in state anxiety at PT relative to BL (median BL 23.5 vs. PT 30.5; *Z*=−2.19, *p*=0.028). As shown in Table 5, at BL, dependent users had significantly higher scores than nondependent users on the BDI and on CAPE total and CAPE-negative symptom frequency scores, and significantly lower GAF and SOFAS scores. At PT, dependent users differed significantly only on the GAF. These data support the evidence in Figure 3 of greater potential efficacy of CBD treatment in dependent users (who may have had more room to move) than in nondependent users, closing the gap between these groups.

**Table 5.** **Significant Symptomatic and Psychological Functioning Differences Between Dependent and Nondependent Users at Baseline and Post-Treatment**

	Baseline			Post-treatment		
	Dependent users	Nondependent users	*p*	Dependent users	Nondependent users	*p*
BDI	4 [0–14]	0.5 [0–2]	0.004^a^	1 [0–9]	0 [0–12]	0.47
CAPE						
Total freq	62.67 (10.95)	54.13 (6.73)	0.045^a^	56.67 (10.04)	55.00 (45–69)	0.69
Neg freq	24.50 (4.81)	20.62 (2.83)	0.036^a^	21.25 (5.29)	22.13 (4.19)	0.70
GAF	77.75 (7.71)	86.25 (6.41)	0.019^a^	79.08 (8.32)	87.50 (6.55)	0.027^a^
SOFAS	77.50 (8.92)	87.25 (4.20)	0.010^a^	79.67 (9.90)	87.75 (5.90)	0.053

Mean (SD) or median [range].

^a^ Significant between-group differences for dependent versus nondependent cannabis users at baseline and post-treatment.

CAPE Total Freq, CAPE total symptom frequency scores; CAPE Neg Freq; CAPE negative symptom frequency scores; Dependent, dependent cannabis-user group (defined as ≥3 on the SDS at baseline); Nondependent, nondependent cannabis user group (defined at <3 on the SDS at baseline).

### BL to PT differences in cognitive performance

Verbal learning and memory performance as measured by the RAVLT were superior at PT relative to BL, with participants recalling more words across the five learning trials and postinterference, but not for delayed recall (Table 4 and Fig. 2). There were no significant time-by-group interactions.

In the AST, while accuracy did not significantly improve, overall median reaction times were faster at PT than BL overall (*p*=0.025) and for incongruent trials (*p*=0.002), with a trend for congruent trials (*p*=0.053; Table 4). The latency difference between congruent and incongruent trials was significantly smaller at PT than BL (*p*=0.009), indicating less variation in performance between trial types at PT due to faster responses in incongruent trials. Importantly, performance improved significantly at PT in trials in which participants were required to switch their attention between response rules, with faster responding at PT (*p*=0.008; Fig. 2)—this variable being the key outcome measure from the AST (Cambridge Cognition Ltd, 2017). Switching cost (the latency difference between switching and nonswitching blocks) was lower at PT than BL (*p*=0.028). There were no time-by-group interactions for any AST variables. Previous research has demonstrated a practice effect on the AST for switch errors and incongruent errors.^48^ No significant change was observed on these variables from BL to PT (both *p*>0.34), arguing against a practice effect in this study, and indeed negative correlations were observed with plasma CBD concentrations, indicating fewer errors PT with higher plasma CBD (*rho* range −0.40 to −0.58, *p* range 0.08 to 0.007).

### Association between plasma CBD concentrations, patterns of cannabis use, and changes in psychological symptoms and cognitive performance

**FIG. 4.**
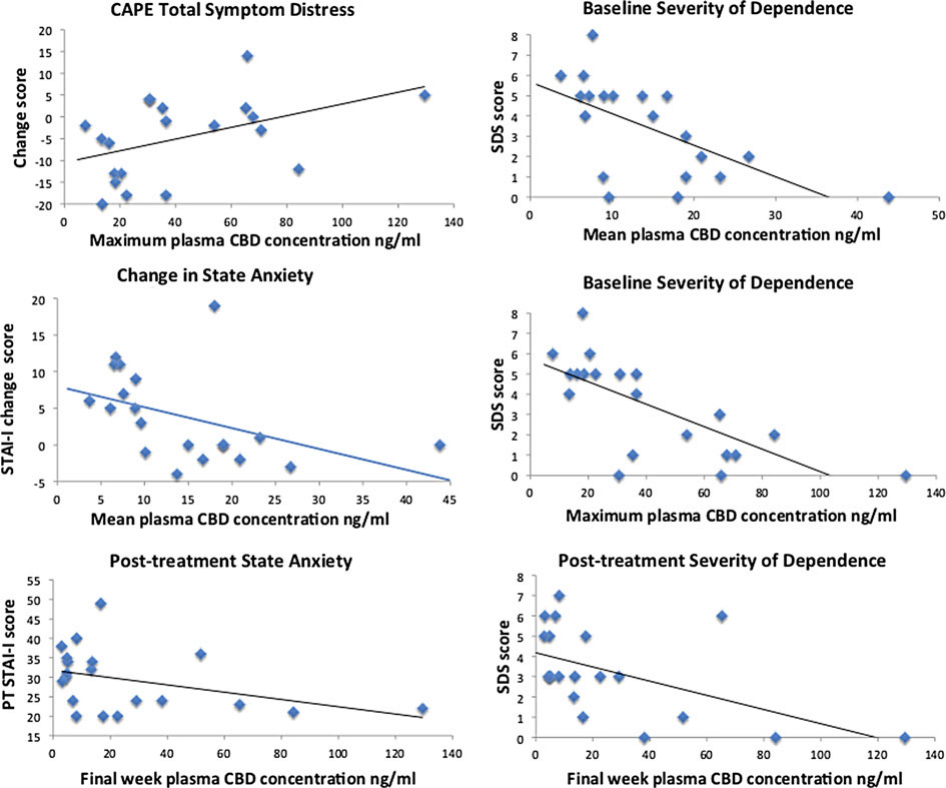
Associations between plasma CBD concentrations, symptoms, and dependence.

Changes in cannabis use patterns, BDI scores, and RAVLT performance were not associated with plasma CBD concentrations. Changes in CAPE total distress (*rho*=0.47, *p*=0.037; Fig. 4), negative symptom frequency (*rho*=0.54, *p*=0.015), and distress scores (*rho*=0.63, *p*=0.003) were significantly correlated with maximum plasma CBD concentrations. CEQ Psychotic change scores correlated with total dose of CBD reported to have been consumed (*rho*=0.51, *p*=0.022), but not with plasma concentrations. Mean, maximum, or final week plasma CBD concentrations were negatively correlated with PT state anxiety and change in state anxiety scores—the higher the average plasma concentration, the greater the *reduction* in state anxiety (*rho* range −0.48 to −0.62, *p* range 0.033 to 0.004) (even though PT state anxiety levels were elevated relative to BL). This implies that higher doses of CBD may alleviate anxiety, but lower doses of CBD may potentially elevate state anxiety (Fig. 4), although this requires replication. No associations were observed for trait anxiety, which was the primary focus of this study, as many confounding factors could account for changes in state anxiety. Both BL and PT severity of cannabis dependence (SDS) scores were negatively correlated with mean, maximum, and final week plasma CBD concentrations—the higher the plasma CBD, the lower the SDS score (*rho* range −0.43 to −0.72, *p* range 0.056 to 0.0003), with stronger associations at BL than at PT (Fig. 4), but change in severity of dependence was not correlated with plasma CBD concentration.

AST switching cost was significantly negatively correlated with plasma CBD concentrations on the day of testing (*rho*=−0.61, *p*=0.006; Fig. 2) (indicating that higher plasma CBD was associated with less variation in latencies between switching and nonswitching trials; i.e., less cost), as was response latency in switching blocks (*rho*=−0.45, *p*=0.051; strengthening after removal of one outlier: *rho*=−0.50, *p*=0.036), which also correlated with mean (*rho=*−0.55, *p*=0.012) and maximum (*rho*=−0.53, *p*=0.016) plasma CBD concentrations. There was no evidence that better functioning people may have taken more capsules, as there were no associations between these AST measures at BL and plasma concentrations attained in the trial. There were no associations between AST measures and hours since last CBD dose, indicating that improved performance was not due to the acute effects of CBD. In contrast, hours since last CBD dose did correlate with postinterference (*rho*=−0.53, *p*=0.029) and delayed recall (*rho=*−0.55, *p*=0.022) on the RAVLT PT, and with change in delayed recall from BL to PT (*rho*=0.50, *p*=0.041), suggesting that acute effects of CBD may contribute to improved memory function.

## Discussion

This is the first study to report the effects of a prolonged course of daily administration of CBD to cannabis users in the community. Ten weeks of 200 mg oral CBD daily was well tolerated, with no side effects during or after completion. In this pragmatic trial, the cannabis users were not seeking treatment and continued their regular pattern of cannabis use during the trial without restriction. Of note, significant reductions in depressive and psychotic-like symptoms were observed, along with improvements in cognition, from BL to the end of treatment.

Caution must be observed in interpreting the results of this open-label trial, as it was not placebo controlled and given the exploratory nature of our analysis. As such, the outcomes may be confounded by a range of bias, expectancy, and practice effects. Nevertheless, the findings suggest that CBD treatment may confer benefits to mental health and cognitive function that are likely CBD treatment specific, as correlations were observed with plasma CBD concentrations. Furthermore, these beneficial outcomes were observed in the context even of ongoing cannabis use. Within a treatment setting for cannabis users seeking to reduce their cannabis use, adjunct use of CBD alongside psychological treatments for cannabis dependence may confer even greater benefits. This premise is based on the following predicates: (1) the cannabis users of this study reported experiencing less euphoria when they smoked cannabis, both subjectively throughout the trial and as measured by the CEQ. While cannabis use in this study neither increased in compensation, nor decreased, in a treatment-seeking sample motivated to reduce or stop using, provided with supportive psychological treatment, the reduced euphoria may facilitate disincentive to continue using; (2) greater beneficial effects of CBD were observed in dependent than nondependent users. This result speaks to the likelihood that CBD confers greater therapeutic effects in a disease state/compromised brain. For example, we recently reported therapeutic neuroprotective effects of CBD in a preclinical model of schizophrenia, where CBD had no effect on cognition or social interaction (the negative symptoms of schizophrenia) in control animals.^49^ Similarly, in this study, cannabis users likely to have a cannabis use disorder (based on SDS cut-off score ≥3) showed significantly greater reduction in symptoms and improved cognition than nondependent users. Interestingly, both severity of dependence on cannabis and AUDIT scores tended to decrease in dependent users only. Dependent users also appeared to absorb or metabolize CBD differently to nondependent users, yet the therapeutic effects of CBD held after controlling for their lower mean plasma CBD concentrations. Further research could look to optimizing CBD delivery and bioavailability, using higher doses of CBD, and exploring mechanistically these differential findings in dependent versus nondependent cannabis users. We acknowledge limitations regarding self-reported medication adherence in terms of interpreting the altered metabolism apparent in dependent users and we were unable to assay CBD metabolites in this study; these would be informative in future prolonged administration studies.

Together with our previous findings suggesting protection of brain harms by CBD,^18^ the current data provide hope that even if cannabis users do not cease to use cannabis in the course of psychological interventions for cannabis dependence, adjunct treatment with CBD may minimize any further harm from continued use. It may be that the greatest efficacy would be achieved with CBD treatment alongside abstinence from cannabis use. Ascertaining the efficacy of CBD treatment in abstinent, dependent users is an important avenue for future research. There was little in the literature to guide the dose of CBD employed in this protocol, and we opted for a conservative/cautious dosing regimen for this first-of-kind study. Further dose-finding studies could manipulate not only the daily dose and its regimen (morning and evening dosing) but also the duration of treatment; we chose 10 weeks of treatment for logistic and feasibility optimization purposes. This was deemed long enough on the basis of other pharmacological clinical trials more generally, to show change in symptoms and cognition, but not so long that significant attrition or nonadherence to trial inclusion criteria might manifest (e.g., excess other drug use). Future studies might also examine a range of other cognitive, clinical and brain functional outcome measures.

Although our findings require replication in a larger sample placebo-controlled trial, the potential for CBD treatment to reduce psychological symptoms and improve cognition in cannabis users, and be further developed as an adjunct to psychological treatments for cannabis dependence, appears very promising.

## Abbreviations Used

AST: Attention Switching Task
BDI: Beck Depression Inventory
BL: baseline
CAPE: Community Assessment of Psychic Experiences
CBD: cannabidiol
CEQ: Cannabis Experiences Questionnaire
CWS: Cannabis Withdrawal Scale
EEG: electroencephalogram
GAF: Global Assessment of Functioning
MRI: magnetic resonance imaging
PT: post-treatment
RAVLT: Rey Auditory Verbal Learning Test
SDS: Severity of Dependence Scale
STAI: State-Trait Anxiety Inventory
THC: Δ^9^-tetrahydrocannabinol
TLFB: Timeline Follow-Back procedure
